# Valorisation of sawdust-based spent mushroom substrate for sustainable xylooligosaccharides production using low-cost crude xylanases from *Aspergillus flavus* KUB2

**DOI:** 10.1080/21501203.2024.2305719

**Published:** 2024-01-31

**Authors:** Surasak Supmeeprom, Anon Thammasittirong, Sukanya Jeennor, Kathawut Sopalun, Sutticha Na-Ranong Thammasittirong

**Affiliations:** a Department of Science and Bioinnovation, Faculty of Liberal Arts and Science, Kasetsart University, Nakhon Pathom, Thailand; b Microbial Biotechnology Unit, Faculty of Liberal Arts and Science, Kasetsart University, Nakhon Pathom, Thailand; c Functional Ingredients and Food Innovation Research Group (IFIG), National Center for Genetic Engineering and Biotechnology (BIOTEC), National Science and Technology Development Agency (NSTDA), Khlong Luang, Thailand

**Keywords:** Spent mushroom substrate, xylooligosaccharides, prebiotic, antioxidant activity, *Aspergillus flavus*, xylan

## Abstract

Spent mushroom substrate (SMS), a lignocellulosic waste after mushroom production is generally discarded without proper management. There is increasing interest in the sustainable transformation of lignocellulosic waste into high-value products. Within this context, the present study investigated the potential of the SMS from the cultivation of *Pleurotus pulmonarius* and *Auricularia auricula* on rubber tree wood sawdust as substrates for xylooligosaccharides (XOS) production. SMS samples from these two edible mushrooms were extracted using alkaline xylan extraction, producing maximum true recovery amounts of xylan in the range 34.61%–37.49% using 18% NaOH at 70 °C for 3 h. Production of XOS from alkaline-extracted xylan from the SMS samples of both mushroom species using economically crude xylanases from *Aspergillus flavus* KUB2 resulted in XOS (X2–X5) production of 241.47–249.04 mg/g, with X3 as the predominant XOS product. The produced XOS had excellent prebiotic activity and 2,2′-diphenyl-1-picrylhydrazyl (DPPH) radical scavenging activity and contained high total phenolic contents. The combined beneficial bioactivities in terms of prebiotic and antioxidant properties suggested that the XOS produced from sawdust-based SMS samples of *P. pulmonarius* and *A. auricula* could be promising ingredients for both food and pharmaceutical applications.

## 1. Introduction

Mushrooms are saprophytic fungi that have a long history of use as human food in various civilisations worldwide. Apart from its nutritional value, mushrooms also contain several bioactive compounds exhibiting health-promoting characteristics, such as antioxidant, anticancer, and immunomodulator properties (Bhambri et al. 2022; Łysakowska et al. 2023). Following the increasing trend towards food consumption with health-beneficial properties, mushrooms have become more attractive as a food item and as an additive in food and pharmaceutical products. Therefore, commercial mushroom cultivation has increased globally, including in Thailand. The rapid growth of the mushroom market has resulted in a large amount of spent mushroom substrate (SMS), consisting of lignocellulosic by-products after harvesting the mushroom sporocarps. The production of 1 kg of fresh mushrooms generates approximately 5 kg of SMS (Mohd Hanafi et al. 2018). Generally, SMS is discarded as waste in landfills or burnt in the field resulting in environmental problems. Only a small amount is converted into low-market-value products, such as biofertiliser, animal feed supplements, and energy feedstock (Grimm and Wösten 2018). Therefore, more efficient utilisation of SMS for high-value product production would not only solve environmental problems but also provide sustainable economic competitiveness by converting lignocellulosic waste to more valuable end products.

Xylooligosaccharides (XOS) are non-digestible oligosaccharides reported to stimulate the growth and activity of beneficial human gut microbiota. In addition, XOS has demonstrated other biological benefits, including anti-inflammatory, antioxidant, and immunomodulator properties (Capetti et al. 2021; Valladares-Diestra et al. 2023). Recently, increasing interest in the health benefits of prebiotics highlighted XOS as a potential functional food ingredient. The global XOS market has been estimated to reach USD 33 million by 2029 (The Market Reports 2023). XOS has generally been recognised as safe (GRAS) certification by the Food and Drug Administration (FDA) in the USA (FDA 2018). Other organisations worldwide have also approved XOS as a safe food ingredient, including the European Food Safety Authority (EFSA) Panel on Dietetic Products, Nutrition and Allergies (NDA) (Turck et al. 2018) and Food for Specified Health Uses (FOSHU) in Japan (Huang et al. 2022). XOS can be produced using the hydrolysis of xylan-rich raw lignocellulosic biomass. Corncob and sugarcane bagasse are the main feedstocks for commercial XOS production (Poletto et al. 2020). To satisfy the growing demand for XOS, various lignocellulosic materials have been investigated as substrates for XOS production, such as wheat bran (Valladares-Diestra et al. 2023), oil palm frond bagasse (Mazlan et al. 2019), mahogany and mango wood sawdust (Rajagopalan et al. 2017), and pistachio shell (Hesam et al. 2020).

Mushrooms can be cultured from different lignocellulosic materials depending on the mushroom species and the local availability of substrates (Mohd Hanafi et al. 2018). Several researchers have reported that SMS has a high hemicellulose content (Rajavat et al. 2020; Seekram et al. 2021; Devi et al. 2023). Despite the great interest in SMS as a xylan source for producing XOS, few studies have been published. Corncob SMS after *Flammulina velutipes* (enokitake) cultivation has been reported as the substrate to produce XOS via hydrothermal treatment (Makishima et al. 2006; Sato et al. 2010). In Thailand, rubber tree (*Hevea brasiliensis*) sawdust is the most common lignocellulosic residue for mushroom cultivation and is mixed with 6% rice bran, 1% CaO, and 0.2% MgSO_4_⋅H_2_O on a dry weight basis of substrate. Recently, we reported the production of XOS from SMS after *Pleurotus ostreatus* cultivation using *Aspergillus flavus* KUB2 crude xylanases with less β-xylosidase activity (Seekram et al. 2021). Xylanase enzyme preparations with low β-xylosidase activity seem promising to reduce xylose formation during XOS production. The present study aimed to further investigate the potential of SMS of two other common edible mushrooms (*Pleurotus pulmonarius* and *Auricularia auricula* that are widely produced on a commercial basis in Thailand) as substrates for XOS production using low-cost crude xylanases from *A. flavus* KUB2. Derived XOS products from the SMS samples of both mushroom species were evaluated for their biological properties. To the best of our knowledge, this is the first report of enzymatic XOS production from sawdust-based SMS of *P. pulmonarius* and *A. auricula* with health-related benefits, including prebiotic and antioxidant properties.

## 2. Materials and methods

### 2.1. Raw materials and compositional analysis

The SMS samples after *P. pulmonarius* and *A. auricula* cultivations and the rubber tree sawdust were provided from a mushroom local farm in Phetchaburi province, Thailand. Initially, the raw materials were dried in sunlight and then in an oven at 60 °C for 48–60 h. The dried biomass samples were determined for their amounts of lignin, α-cellulose, and holocellulose, according to the methods of the Technical Association of the Pulp and Paper Industry (TAPPI) as detailed in TAPPI T222-om02 (TAPPI 2002), TAPPI T203-om-93 (TAPPI 1993), and Browning (1967), respectively.

### 2.2. Xylan extraction

The xylan was extracted from the SMS following the method described by Seekram et al. (2021) with some modifications. The SMS samples were soaked in a NaOH solution (12%, 14%, 16%, or 18%) using a solid-to-liquid ratio of 1:10 at ambient temperature with shaking at 150 r/min for 30 min and then incubated at 70 °C for 3 h. The solution was filtered through a cheesecloth to remove the solid residue and the soluble xylan was acidified at pH 5.0. The xylan was precipitated in 3 volumes of 95% ethanol. After settling overnight at 4 °C, the precipitated xylan was collected using centrifugation at 12,000 ×*g* for 20 min, dried at 50–60 °C until the constant weight was recorded, and then powdered using a grinder. The true recovery of xylan was calculated using the formula (Banerjee et al. 2019): True recovery (%) = (Dry weight of extracted xylan/Dry weight of sample) × 100.

### 2.3. Fourier-transform infrared spectroscopy (FT-IR) analysis

The extracted xylan from the SMS and commercial beechwood xylan were subjected to FT-IR analysis (Vertex 70, Bruker; Ettlingen, Germany). The FT-IR spectrophotometer was operated at a spectral range of 4,000–400 cm^−1^, with a spectral resolution of 4 cm^−1^.

### 2.4. Preparation of crude xylanases

A crude xylanases enzyme was produced using *A. flavus* KUB2 (Namnuch et al. 2021). For the xylanase production, sugar cane bagasse was used as the carbon source under submerged fermentation at 30 °C with shaking at 150 r/min for 5 days, as described by Namnuch et al. (2021). At the end of fermentation, the culture broth was centrifuged at 12,000 ×*g* for 15 min at 4 °C and used as crude xylanases. The xylanase activity was assayed according to Bailey et al. (1992). One unit (U) of enzyme activity was defined as the amount of enzyme required to release 1 μmol of xylose per minute.

### 2.5. *Characterisation of* A. flavus *KUB2 crude xylanases*

The optimum pH was determined by measuring the xylanase activity at different values of pH (0.05 mol/L sodium citrate buffer for pH 4.0–6.0 and 0.05 mol/L Tris-HCl buffer for pH 7.0–10.0) at 50 °C for 5 min. The pH stability was determined by assaying the remaining enzyme activity after incubating in different buffers (pH 4.0–10.0) without substrates at 4 °C for 1 h.

The optimum temperature was determined by measuring the xylanase activity at different values of temperature (30–75 °C) for 5 min at the optimum pH. The thermal stability was determined by measuring the remaining enzyme activity after incubation at different temperatures (30–75 °C) at the optimum pH without substrate for 1 h (Ketsakhon et al. 2023).

### 2.6. Enzymatic XOS production

The production of XOS from the alkaline-extracted xylan of SMS was performed using crude xylanases from *A. flavus* KUB2, according to Seekram et al. (2021), with slight modification. The reaction mixture contained 1% xylan and 20 U/g xylan of crude xylanases in 50 mmol/L sodium citrate buffer at pH 5.0. Enzymatic hydrolysis was performed at 55 °C for 24 h. The non-hydrolysed xylan was precipitated in 3 volumes of ethanol and then removed using centrifugation at 12,000 ×*g* and 4 °C for 15 min. Ethanol was removed from the soluble XOS in the liquid fraction using rotary evaporation.

### 2.7. XOS analysis

The XOS was quantified using high-pressure liquid chromatography (HPLC, Ultimate 3000, Thermo Fischer Scientific; Franklin, MA, USA) equipped with a refractive index detector and a Shodex^TM^ SC1011 column (Resonac America, Inc.; New York, NY, USA). The hydrolysed samples passed through a 0.2 µm sterile filter and were then subjected for analysis. The column was run in isocratic mode using distilled water type I as the mobile phase at 80 °C at a flow rate of 0.6 mL/min for 20 min. Quantitative analysis of XOS was performed using a calibration curve of XOS standards (Megazyme; Wicklow, Ireland).

### 2.8. In vitro *fermentation of XOS by probiotic bacteria*

The probiotic activity of XOS was performed by studying the effect on the growth of the probiotic bacteria, *Lacticaseibacillus casei* (formerly *Lactobacillus casei*) TISTR 1463 and *Lactiplantibacillus plantarum* (formerly *Lactobacillus plantarum*) TISTR 2075, obtained from the Thailand Institute of Scientific and Technology (TISTR), Thailand, as described by Seekram et al. (2021). Each probiotic bacterium was inoculated in de Man Rogosa and Sharpe (MRS) broth at 37 °C for 24 h. The OD_600_ of the pre-culture was adjusted to 1.0 and then 2% (v/v) of adjusted cultured was inoculated in MRS broth without any carbon source, supplemented with xylose or XOS from the SMS samples of either *P. pulmonarius* or *A. auricula* as carbon sources. The cultures were incubated at 37 °C in an anaerobic jar under static conditions for 24 h. The growth of probiotic cultures was determined using a viable cell count on the MRS plates.

### 2.9. 2,2-diphenyl-1-picrylhydrazyl (DPPH) radical scavenging activity assay

The DPPH method was performed according to Bian et al. (2013) with slight modification. In brief, 1 mL of XOS sample was added to 1 mL of 0.1 mmol/L DPPH in ethanol. The mixture was vortexed and then incubated at room temperature in the dark for 2 h. Next, the absorbance of the reaction mixture was measured at 517 nm. The control was carried out using deionised water and DPPH solution, while ethanol was used as the blank. The DPPH radical scavenging activity was calculated using the following equation:

DPPH scavenging activity (%) = (1 - Absorbance of sample/Absorbance of control) × 100

The concentration of XOS required to quench 50% of the initial DPPH radical was defined as the IC_50_ value.

### 2.10. Determination of total phenolic content

The total phenolic content was determined using the Folin-Ciocalteu reagent following the method described by Wu et al. (2016). Briefly, 0.3 mL of XOS sample was added to 1.5 mL of 0.2 mol/L Folin-Ciocalteu reagent and incubated at room temperature for 5 min, then it was followed by adding 1.2 mL of 0.7 mol/L sodium carbonate solution. After incubation for 2 h in the dark at room temperature, the absorbance was measured at 760 nm. The total phenolic contents were determined from the linear equation of a standard curve obtained from gallic acid. The results were expressed in milligrams of gallic acid equivalent per milligram of XOS (mg GAE/mg).

## 3. Results and discussion

### 3.1. Raw material chemical composition

The results of the analysis of the cellulose, hemicellulose, and lignin contents of the SMS after *P. pulmonarius* and *A. auricula* cultivations and the rubber tree wood sawdust (control) are shown in Table 1. The results demonstrated that the cultivation of these two edible mushrooms altered the chemical composition of rubber tree wood sawdust used as mushroom substrate. *P. pulmonarius* and *A. auricula* belong to the Basidiomycota. These basidiomycetes are considered as primary decomposers that release extracellular ligninolytic and hydrolytic enzymes to degrade the carbohydrate in plant cell walls during mushroom growth and production (Grimm and Wösten 2018). This resulted in a reduction in the lignocellulose contents of SMS. The chemical composition of the SMS varies depending on the mushroom species and the type of lignocellulosic biomass used as cultivation substrate for mushroom production (Devi et al. 2023), with the type of biomass substrate having the main effect on the residue contents of lignocellulosic composition (Bae et al. 2006). The rubber tree wood sawdust mushroom substrate after *P. pulmonarius* and *A. auricula* cultivations contained higher hemicellulose contents compared to the paddy straw SMS of *Volvariella volvacea*, the wheat straw SMS of *Calocybe indica*, *Pleurotus florida*, and *P. ostreatus*, and the cotton waste SMS of *P. ostreastus* (Table 1). The hemicellulose values of SMS in this study were similar to those of the raw material for commercial XOS production, cane bagasse (23%–28%) (Jayapal et al. 2013; Thammasittirong et al. 2023) and higher than for other conventional biomass sources, such as spent malt (21.8%), soybean hull (19.5%), and rice husk (11.2%) (da Silva Menezes et al. 2017). The chemical composition of lignocellulose materials may vary due to plant species, cultivation conditions, and the determination method (Kasiri and Fathi 2018; Thammasittirong et al. 2023). The considerably higher hemicellulose content in the SMS samples of *P. pulmonarius* and *A. auricula* reflected their potential as economically sustainable substrates for XOS production.

**Table 1.** Chemical composition (% dry basis) of rubber tree wood sawdust and spent mushroom substrate (SMS).

Substrate	Cellulose (%)	Hemicellulose (%)	Lignin (%)	Reference
Rubber tree wood sawdust	40.77 ± 0.30^a^	31.61 ± 0.07^a^	23.04 ± 0.217^a^	This study
SMS of *Pleurotus pulmonarius* (sawdust substrate)	31.92 ± 1.06^c^	27.78 ± 1.32^b^	19.95 ± 0.05^b^	This study
SMS of *Auricularia auricula* (sawdust substrate)	33.45 ± 0.25^b^	29.40 ± 1.90^b^	19.56 ± 0.18^b^	This study
SMS of *P. ostreatus* (sawdust substrate)	32.38 ± 0.08	25.02 ± 0.07	22.75 ± 0.01	Seekram et al. (2021)
SMS of *P. florida* (wheat straw substrate)	37.5 ± 1.1	18.6 ± 0.5	20.5 ± 1.2	Rajavat et al. (2020)
SMS of *P. ostreatus* (wheat straw substrate)	30.5	17.8	19.4	Fang et al. (2020)
SMS of *Volvariella volvacea* (paddy straw substrate)	41.05 ± 1.03	20.79 ± 2.39	10.02 ± 1.81	Devi et al. (2023)
SMS of *Calocybe indica* (wheat straw substrate)	39.47 ± 1.02	21.22 ± 2.79	13.25 ± 0.38	Devi et al. (2023)
SMS of *P. ostreatus* (cotton waste substrate)	44.1	3.1	16.7	Bae et al. (2006)

Different lowercase superscripts in each column indicate significant (*P* < 0.05) differences among rubber tree wood sawdust, SMS of *Pleurotus pulmonarius* and SMS of *Auricularia auricula*. Values represent mean ± standard deviation from three independent experiments.

### 3.2. Xylan extraction

In lignocellulosic biomass, hemicellulose closely binds to cellulose through hydrogen bonds and links to lignin through covalent bonds to form a lignin-carbohydrate complex (Zoghlami and Paës 2019). Alkaline reagents cause cellulose swelling and decrease crystallinity, effectively delignifying lignocellulose and breaking down the chemical connection between lignin and hemicellulose, which make the alkaline extraction method a favourite for xylan extraction (Khangwal et al. 2020). In the present study, xylan from the SMS samples of *P. pulmonarius* and *A. auricula* was extracted using 12%, 14%, 16%, or 18% NaOH at 70 °C for 3 h. The highest NaOH concentration (18%) was able to recover the highest xylan from the SMS of *P. pulmonarius* (34.61%) and *A. auricula* (37.49%), as shown in Table 2. Seekram et al. (2021) reported the extraction of xylan from the SMS of *P. ostreatus* using concentrations of NaOH in the range of 1–4 mol/L (~4%–16%) at 50 °C for 3 h, where the highest true recovery of xylan (20.76%) was observed under the highest concentration of NaOH. Jnawali et al. (2018) reported that increasing the concentration of NaOH resulted in a higher true yield of xylan, with the highest true recovery of coconut husk xylan (24%) being obtained with 20% NaOH at 121 °C for 1 h. Increasing alkaline concentration leads to a higher true recovery of xylan. This may have been due to the linkage bonds between hemicellulose and lignin being more broken, resulting in the greater dissolution of the hemicellulose (Jin et al. 2019). However, extraction with a high concentration of the alkaline solution may lead to the high salt formation during the neutralisation step (Lehuedé et al. 2023). There should be further investigation of salt formation and other impurities in the extracted xylan from the SMS samples and optimisation of the xylan extraction to improve the quality and yield of xylan.

**Table 2.** Alkaline extraction of xylan from SMS of *Pleurotus pulmonarius* and *Auricularia auricula*.

NaOH concentration (%)	True recovery of xylan (%)	
	SMS of *P. pulmonarius*	SMS of *A. auricula*
12	17.43 ± 0.47^d^	15.85 ± 0.00^d^
14	22.81 ± 1.97^c^	18.45 ± 0.47^c^
16	27.28 ± 0.50^b^	26.84 ± 2.16^b^
18	34.61 ± 1.62^a^	37.49 ± 1.39^a^

Different lowercase superscript letters in a column indicate significant (*P* < 0.05) differences among NaOH concentrations. Values represent mean ± standard deviation from three independent experiments.

### 3.3. FT-IR analysis

**Figure 1.**
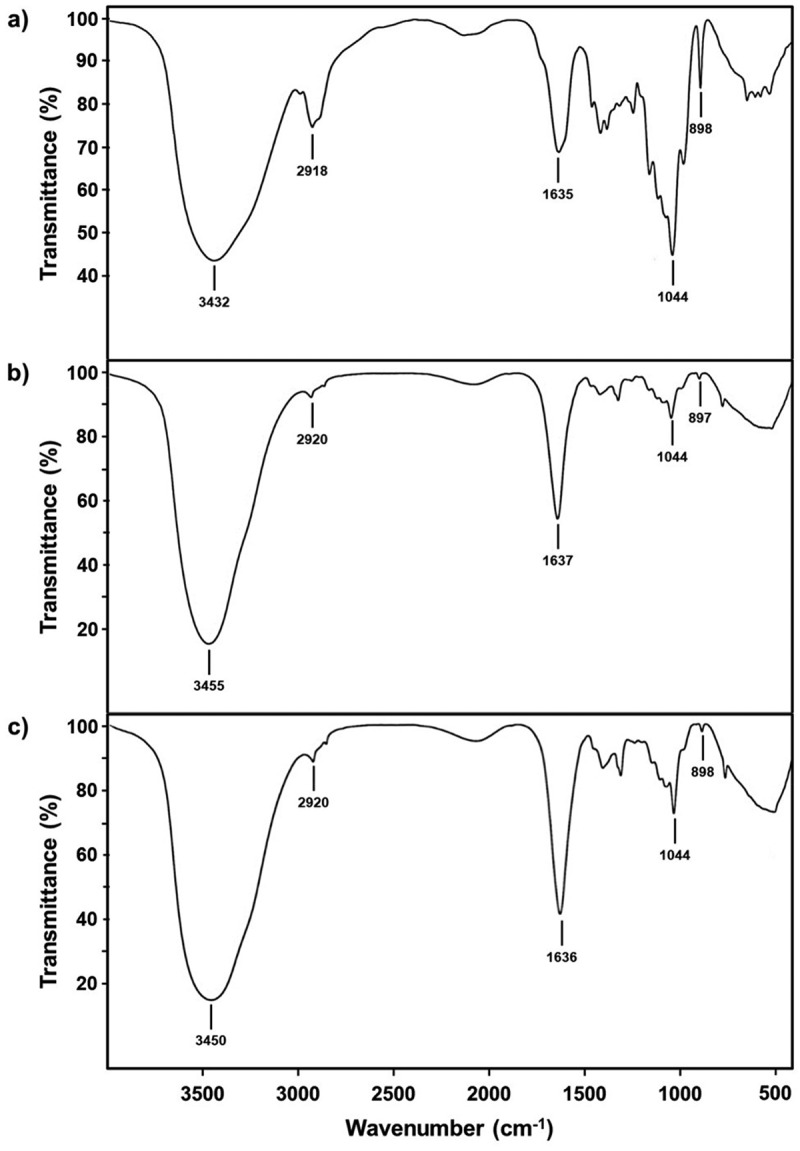
FT-IR spectra of commercial beechwood xylan (a), alkaline-extracted xylan from SMS of *Pleurotus pulmonarius* (b), and alkaline-extracted xylan from SMS of *Auricularia auricula* (c).

FT-IR spectroscopic analysis was performed to determine the structural characteristics of the SMS xylans. The FT-IR spectra for the extracted xylan samples from *P. pulmonarius* and *A. auricula* and commercial beechwood xylan are shown in Figure 1. The broadband in the region 3,600–3,100 cm^−1^ was related to the hydroxyl (OH) groups in xylan. The band around 2,920–2,910 cm^−1^ was ascribed to C-H stretching (Egües et al. 2010; Banerjee et al. 2019). The bands in the range 1,637–1,635 were assigned to the water bending mode (Oliveira et al. 2010). The band observed at 1,044 cm^−1^ was associated with hemicellulose and attributed to C-O, C-C stretching, or C-OH bending in xylan (Oliveira et al. 2010; Banerjee et al. 2019). The band around 898–897 cm^−1^ represented the β-glycosidic linkages between the sugar units in hemicellulose (Gil-Ramirez et al. 2018; Sharma et al. 2020).

The FT-IR spectra of the SMS xylans demonstrated the presence of common functional groups similar to the standard xylan from commercial beechwood, suggesting the successful extraction of the xylan fraction from the SMS samples of *P. pulmonarius* and *A. auricula*.

### 3.4. *Characterisation of* A. flavus *KUB2 crude xylanases*

**Figure 2.**
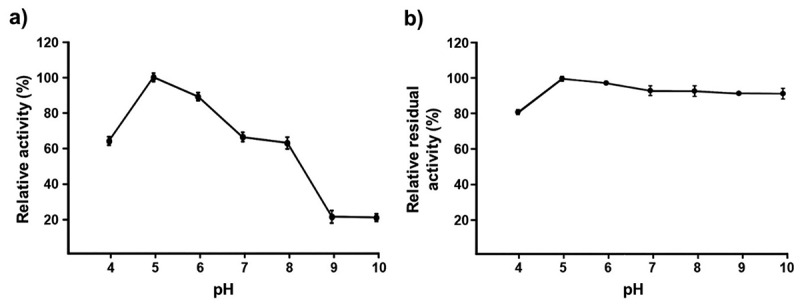
Effect of pH on xylanase activity (a) and stability (b).

The cost of the enzyme is one of the major factors determining the economics of a process. Seekram et al. (2021) reported the successful use of low-cost crude xylanases without any purification from *A. flavus* KUB2 to obtain XOS from rubber tree sawdust waste after *P*. *ostreatus* cultivation. To further characterise the enzyme, the effect of pH (4.0–10.0) and temperature (30–75 °C) on the levels of crude xylanase activity and stability were determined to identify the optimum conditions for enzyme catalysis. The results showed that the optimal pH for the crude xylanases was pH 5.0 (Figure 2a). The enzyme retained over 60% of the original activity at pH levels of 4.0, 6.0, 7.0, and 8.0 and significantly decreased its activity at pH 9.0–10.0, retaining only 25.19%–25.59%. Similar to *A. flavus* KUB2, several other fungal xylanases showed a similar optimum range of pH 5.0–6.0 and less activity at pH > 8.0, for example *Aspergillus fumigatus* N2 (Lin et al. 2017), *Aspergillus foetidus* MTCC 4898 (Shah and Madamwar 2005), and *Penicillium crustosum* ABQ1 (Espinoza-Abundis et al. 2023). The crude xylanases of *A. flavus* KUB2 were highly stable over a broad pH range of 4.0–10.0, retaining 80.88%–98.12% of its activity (Figure 2b). Its stability over a wide pH range is favourable for different industrial applications involving acidic, neutral, and alkaline conditions.

**Figure 3.**
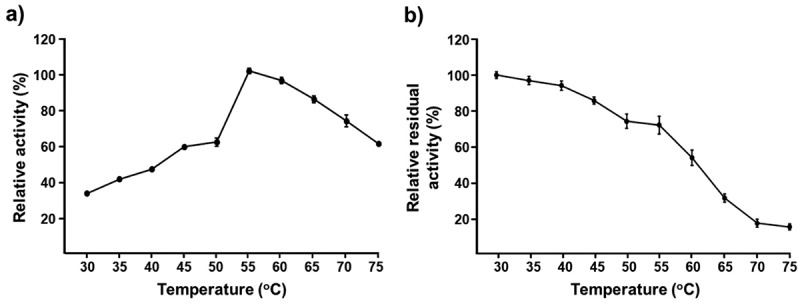
Effect of temperature on xylanase activity (a) and stability (b).

To investigate the influence of temperature on xylanase activity, crude xylanases enzyme was incubated at temperatures 30–75 °C. The enzyme activity increased with increased temperature, with the optimal activity at 55 °C (Figure 3a). Enzyme activity levels of 97.16%, 86.24%, and 74.54% were retained when the temperature increased to 60 °C, 65 °C, and 70 °C, respectively. Xylanase enzymes with similar temperature optima have been reported from various fungi, for example, *A. flavus* CICC No. 2476 (Chen et al. 2019), *A. fumigatus* M51 and *Trichoderma reesei* CCT 2768 (Carvalho et al. 2015).

Regarding thermal stability, the crude xylanases enzyme was stable in the 30–55 °C range, retaining 71.44%–99.35% enzyme activity (Figure 3b). However, the enzyme had residual activity below 20% at 70–75 °C after 1 h. Xylanase from *A. foetidus* MTCC4898 was very stable at 30–40 °C, while the residual activity was less than 20% at 60 °C within 30 min (Shah and Madamwar 2005). Xylanase of *Cerrena unicolor* VKM F-3196 retained 50% and 35% of the initial activity after 1 h at 50 °C and 60 °C, respectively (Belova et al. 2014). Xylanase of *Paecilomyces variotii* retained 80% of the original activity after incubation for 30 min at 60 °C (Abdella et al. 2021). The stability of the pH and temperature of xylanase is markedly dependent on fungal species. Such differences might be due to the effect of different enzyme mixtures and/or post-translational modifications of xylanase during the excretion process (Sá-Pereira et al. 2002; Lafond et al. 2011).

### 3.5. Enzymatic XOS production

**Figure 4.**
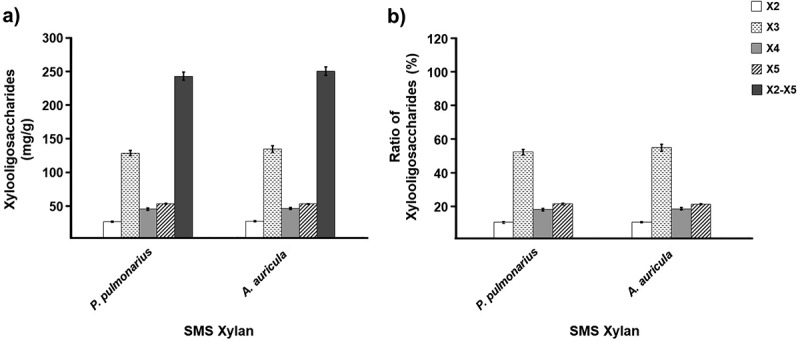
XOS production from SMS xylan of *Pleurotus pulmonarius* and *Auricularia auricula* using crude xylanases of *A. flavus* KUB2. XOS yield (a) and percentage ratio of XOS with DP 2–5 (b).

In the present study, the alkaline-extracted xylans of the SMS after *P. pulmonarius* and *A. auricula* cultivations were subjected to enzymatic hydrolysis using the economically crude xylanases produced in-house from *A. flavus* KUB2 with a xylanase activity of 2,354.38 U/g. Enzymatic hydrolysis of SMS xylan from both resources produced total XOS (X2–X5) in the range of 241.47–249.04 mg/g, of which xylotriose (X3) was a major product, yielding 126.00–132.17 mg/g (~52.18%–53.07% of XOS), as shown in Figure 4. The non-detection of xylose in the hydrolysate may have been due to less β-xylosidase activity of *A. flavus* KUB2 (Seekram et al. 2021). The XOS yields obtained from pure xylan extracted from mahogany and mango wood sawdust samples hydrolysed with purified xylanase from *Clostridium* strain BOH3 were 572 mg/g and 504 mg/g, respectively (Rajagopalan et al. 2017). The enzymatic mixtures with commercial endo-xylanase (*Aspergillus niger*), commercial α-L-arabinofuronosidase (*A. niger*) and commercial feruloyl esterase (*Clostridium thermocellum*) hydrolysed xylan extracted from sugarcane straw and coffee husk produced XOS (X2–X6) with yields of 205 mg/g and 169 mg/g, respectively, with X2 as the dominant product (Ávila et al. 2020). Production of XOS from banana pseudostem xylan using *Aspergillus versicolor* xylanase produced XOS with X5/X6 as the major product (de Freitas et al. 2021). Crude *Pichia stipitis* xylanase hydrolysed sugarcane bagasse xylan and produced X3 as the main component (Bian et al. 2013).

The amount of the obtained XOS and the range in the degree of polymerisation (DP) could be attributed to the difference in the xylanase source and its specificity, lignocellulosic composition of the biomass, xylan structure, and the production conditions, including xylan extraction and enzymatic hydrolysis (Rajagopalan et al. 2017; Mazlan et al. 2019). Therefore, comparison among different reports must take such factors into account in the interpretation of XOS production. The XOS with a low DP (X2–X6), especially X2 and X3, is highly recommended for food-related applications because beneficial probiotic bacteria are more easily degraded and utilised (Rogoski et al. 2022; Yegin 2023). In the present study, the in-house xylanase produced from *A. flavus* KUB2 showed the ability to release short-chain XOS (X2–X5), predominantly X3, from the extracted xylans of the SMS samples of both mushroom species. These traits indicated the potential of the SMS xylans and crude xylanases from *A. flavus* KUB2 as substrate and enzyme for economically sustainable XOS production. Optimisation of enzymatic hydrolysis is required in further studies to maximise the production of XOS with a certain DP of X2–X3.

### 3.6. In vitro *fermentation of XOS using probiotic bacteria*

**Figure 5.**
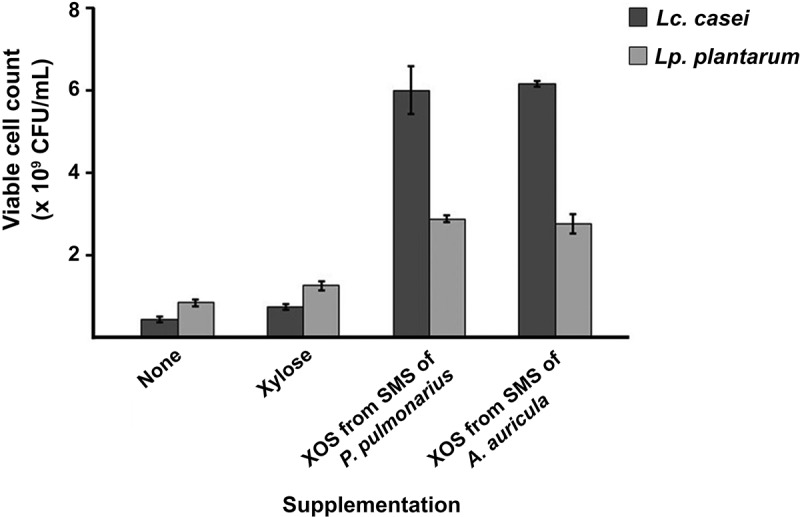
*Lacticaseibacillus casei* TISTR 1463 and *Lactiplantibacillus plantarum* TISTR 2075 growth in MRS media without sugar supplementation and MRS media supplemented with xylose and XOS from SMS of *Pleurotus pulmonarius* and *Auricularia auricula*as carbon source.

The functional potential of the XOS produced from the SMS samples of *P. pulmonarius* and *A. auricula* as a prebiotic was investigated based on the growth-promoting abilities of two known probiotic bacteria: *Lc. casei* TISTR 1463 and *Lp. plantarum* TISTR 2075. As shown in Figure 5, the influence of XOS on growth stimulation was higher than for either xylose or media without sugar supplement. Similar results were reported by de Freitas et al. (2021), Kaur et al. (2019), and Seekram et al. (2021). XOS derived from the SMS samples of both mushroom species had higher growth stimulatory effects on *Lc. casei* TISTR 1463 compared to *Lp. plantarum* TISTR 2075. The difference in the growth patterns of the probiotic bacteria was probably a result of their ability to metabolise XOS. Efficient and complete degradation of XOS depends on the xylanolytic enzyme systems of probiotic bacteria, which seem to be strain-specific. *Levilactobacillus brevis* (formerly *Lactobacillus brevis*) FS2.1 produced xylanase and cell-associated β-xylosidase. *Lp. plantarum* FS46.3 produced xylanase and intracellular β-xylosidase, whereas *Lp. plantarum* FS48.3 could produce only xylanase enzyme (Kanpiengjai et al. 2022). Iliev et al. (2020) reported the presence of intracellular β-xylosidase in *Lv. brevis* S27, *Lp. plantarum* S26, and *Lactobacillus sakei* S16. *Bifidobacterium adolescentis*, *Bifidobacterium infantis*, and *Bifidobacterium bifidum* produced intracellular β-xylosidase and α-arabinosidase, while xylanase, α-glucuronidase and acetyl xylan esterase were not detected (Zeng et al. 2007). In the present study, the excellent probiotic activity of the derived XOS from the SMS samples of *P. pulmonarius* and *A. auricula* using crude xylanases enzyme from *A. flavus* KUB2 revealed the feasibility of an effective prebiotic for commercial food applications.

### 3.7. Antioxidant activity and total phenolic content of XOS

**Figure 6.**
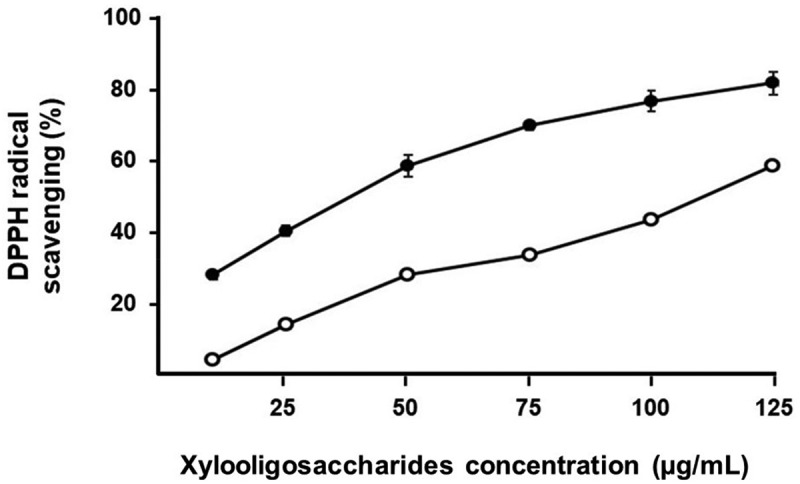
Antioxidant activity against DPPH of XOS from SMS xylan of *Pleurotus pulmonarius* (open symbol) and *Auricularia auricula* (filled symbol).

DPPH assay is a rapid, easy and accurate method widely used to evaluate the free radical scavenging ability of natural compounds (Mwangi et al. 2022). The present results of the DPPH assay demonstrated that the derived XOS from the enzymatic hydrolysis of alkaline-extracted xylan from the SMS samples of *P. pulmonarius* and *A. auricula* using crude xylanases enzyme exhibited scavenging capabilities in a concentration-dependent manner (Figure 6). The XOS from the SMS of *A. auricular* had higher antioxidant activity compared to the XOS from the SMS of *P. pulmonarius*. At a minimal concentration of 10 μg/mL, the XOS from the SMS of *A. auricula* exhibited DPPH inhibition of 26.40% and a gradual increase to a maximum of 80.00% at 125 μg/mL, while the XOS from the SMS of *P. pulmonarius* showed inhibition of 2.74% and 57.05%, respectively, at the same concentrations. The IC_50_ concentrations of the XOS from SMS xylan of *P. pulmonarius* and *A. auricula* were 112.32 μg/mL and 47.89 μg/mL, respectively (Table 3). Veenashri and Muralikrishna (2011) used the DPPH method to study the antioxidant activity of XOS mixtures obtained from rice, ragi, wheat, and maize bran. Among these XOS mixtures, ragi XOS had an effective antioxidant activity of about 12% at a minimum threshold concentration of 10 μg/mL, with its highest activity being 70% at a concentration of 60 μg/mL. The XOS mixture obtained by enzymatic hydrolysis of xylan extracted from empty fruit bunches showed the strongest inhibitory effect against the DPPH radical of 71.36% at 100 μg/mL, with its IC_50_ value being approximately 50 μg/mL (Khangkhachit et al. 2021). Based on the DPPH assay, the IC_50_ values of XOS from the enzymatic hydrolysis of sugarcane bagasse (Bian et al. 2013), corncob (Gowdhaman and Ponnusami 2015), and spray-dried corncob XOS carried by gum Arabic (Zhang et al. 2019) were 620 μg/mL, 1,000 μg/mL, and 1,126 μg/mL, respectively. Unfortunately, to date, no known literature has reported the results of the antioxidant activity from XOS derived from SMS to compare with the present study.

**Table 3.** Antioxidant activity and total phenolic content of XOS from SMS xylan of *Pleurotus pulmonarius* and *Auricularia auricula*.

XOS	Antioxidant methods	
	DPPH (IC_50_ values) (μg/mL)	Total phenolic content (mg GAE/mg)
SMS of *P. pulmonarius*	112.32 ± 2.08^a^	0.34 ± 0.01^b^
SMS of *A. auricula*	47.89 ± 2.38^b^	0.45 ± 0.02^a^

Different lowercase superscript letters in a column indicate significant (*P* < 0.05) differences between SMS sources. Values represent mean ± standard deviation from three independent experiments.

Phenolic compounds have antioxidant properties due to their hydrogen donation or electron donation abilities to scavenge free radicals (Zhang et al. 2019; Ma et al. 2023). In the present study, the total phenolic contents of the produced XOS from the SMS samples of *P. pulmonarius* and *A. auricula* were 0.34 mg GAE/mg and 0.45 mg GAE/mg, respectively (Table 3). These results suggested that the total phenolic compounds found in the SMS-generated XOS products may have contributed to their antioxidant activity in the DPPH assay. According to Shen et al. (2016), phenolic compounds were binding on xylans during xylan extraction from cornstover. Xylan-derived oligosaccharides from the bran of rice, ragi, wheat, and maize were observed with derivatives of cinnamic acid, namely, ferulic acid and *p*-coumaric acid, and with derivatives of benzoic acid, namely, vanillic acid, gallic acid and syringic acid, as bound phenolic acids (Veenashri and Muralikrishna 2011). Boonchuay et al. (2021) reported phenolic compounds in corncob XOS. SMS consists of residual lignocellulosic substrates, and mushroom mycelia and also contains bioactive compounds, including phenolic acids (Mohd Hanafi et al. 2018; Łysakowska et al. 2023), with their profile and content varying according to the mushroom species and cultivation conditions (Łysakowska et al. 2023). The phenolic compounds from mushroom production and lignocellulosic lignin presenting in SMS may bind to xylan in the extraction process and have antioxidant properties. The antioxidant activity of a phenolic compound is related to its concentration and structure (Veenashri and Muralikrishna 2011; Ma et al. 2023). Therefore, the higher antioxidant capacity of the XOS derived from the SMS of *A. auricula* may have been due to its higher total phenolic value compared to that of the XOS derived from the SMS of *P. pulmonarius*. In addition, the SMS of *P. pulmonarius* and *A. auricula* may contain different types of phenolic compounds. To our knowledge, this is the first report on the prebiotic and antioxidant activities of the XOS from the sawdust-based SMS of two edible mushrooms in the *Pleurotus* and *Auricularia* genera. The results suggested that the XOS produced from the SMS samples of *P. pulmonarius* and *A. auricula* could benefit the food and pharmaceutical industries.

## 4. Conclusions

The present study demonstrated valorisation of SMS from *P. pulmonarius* and *A. auricula* as a promising sustainable substrate for XOS production using crude xylanases produced in-house from *A. flavus* KUB2. The XOS derived from the SMS samples of both mushroom species highlighted their potential to promote the growth of probiotic bacteria and to exhibit strong DPPH radical scavenging activity. The generated XOS with prebiotic activity and antioxidant activity, as well as a good total phenolic content suggested it has attractive advantages for use in food and pharmaceutical-related applications. The results of the present study should support a more sustainable economy by adding value to this underutilised material biomass from the mushroom industry.
